# A standardized clinical pathway for hip fracture patients is associated with reduced mortality: data from the Norwegian Hip Fracture Register

**DOI:** 10.1007/s41999-023-00788-9

**Published:** 2023-04-26

**Authors:** Tuva Elisabeth Garre-Fivelsdal, Jan-Erik Gjertsen, Eva Dybvik, Marit Stordal Bakken

**Affiliations:** 1 University of Bergen, Bergen, Norway; 2 The Norwegian Hip Fracture Register, Department of Orthopedic Surgery, Haukeland University Hospital, Bergen, Norway; 3 Department of Clinical Medicine, University of Bergen, Bergen, Norway; 4 Department of Clinical Sciences, University of Bergen, Bergen, Norway

**Keywords:** Hip fracture, Mortality, Standardized clinical pathway, Interdisciplinary treatment

## Abstract

**Purpose:**

A standardized clinical pathway is recommended for hip fracture patients. We aimed to survey standardization of treatment in Norwegian hospitals and to investigate whether this affected 30-day mortality and quality of life after hip fracture surgery.

**Methods:**

Based on the national guidelines for interdisciplinary treatment of hip fractures, nine criteria for a standardized clinical pathway were identified. A questionnaire was sent to all Norwegian hospitals treating hip fractures in 2020 to survey compliance with these criteria. A standardized clinical pathway was defined as a minimum of eight criteria fulfilled. Thirty-day mortality for patients treated in hospitals with and without a standardized clinical pathway was compared using data in the Norwegian Hip Fracture Register (NHFR).

**Results:**

29 out of 43 hospitals (67%) answered the questionnaire. Of these, 20 hospitals (69%) had a standardized clinical pathway. Compared to these hospitals, there was a significantly higher 30-day mortality in hospitals without a standardized clinical pathway in the period 2016–2020 (HR 1.13, 95% CI 1.04–1.23; *p* = 0.005). 4 months postoperatively, patients treated in hospitals with a standardized clinical pathway and patients treated in hospitals without a standardized clinical pathway reported an EQ-5D index score of 0.58 and 0.57 respectively (*p* = 0.038). Significantly more patients treated in hospitals with a standardized clinical pathway were 4 months postoperatively able to perform usual activities (29% vs 27%) and self-care (55% vs 52%) compared to hospitals without a standardized clinical pathway.

**Conclusion:**

A standardized clinical pathway for hip fracture patients was associated with reduced 30-day mortality, but no clinically important difference in quality of life compared to a non-standardized clinical pathway.

## Key summary points

**Aim:**

To survey standardization of treatment in Norwegian hospitals and to investigate whether this affected 30-day mortality and quality of life after hip fracture surgery.

**Findings:**

Only two out of three hospitals had a standardized clinical pathway for hip fracture patients. A standardized clinical pathway for hip fracture patients was associated with reduced 30-day mortality and higher functional level.

**Message:**

A standardized clinical pathway should be implemented at all hospitals treating hip fracture patients.

## Introduction

In Norway, 9000 patients are treated for a hip fracture annually [1]. A hip fracture is a serious event for the patient, with major consequences for mobility, independency, and cognition [2–4]. The mean age of hip fracture patients in Norway is 82 years and 71% are women [5]. The 1-year mortality has been reported to be 24% and the 5-year mortality 60% [6]. Hip fracture patients is a very resource-demanding group for the healthcare system and the number of hip fracture patients is expected to increase in the future [1]. The surgical treatment of hip fractures has changed significantly in recent decades. Femoral neck fractures are now mainly operated with prostheses, and intramedullary nails are increasingly used for unstable trochanteric and subtrochanteric fractures [6]. Further improvement of hip fracture care lies in the perioperative treatment. The key to improve perioperative treatment is systematic care processes, quality registration, and interdisciplinarity.

The Norwegian guidelines for interdisciplinary treatment of hip fractures were published in 2018 [1]. Still, geriatricians are scarce, and much work remains to establish an interdisciplinary orthogeriatric service at many Norwegian hospitals. In the UK, specific criteria for interdisciplinary treatment of hip fractures have been defined in Best Practice Tariff [7]. The Norwegian guidelines do not contain any specific criteria or quality indicators that must be met, but provides recommendations on organization, staffing, and treatment standards. In Norway, many hospitals have adopted a so-called “fast-track” model, where hip fracture patients are admitted directly at the orthopedic ward rather than in the emergency department. The purpose of such a model is to streamline the course of treatment and to shorten the waiting time for surgery. The Norwegian Hip Fracture Register (NHFR) collects data on all hip fracture operations in Norway and monitors the treatment offered to hip fracture patients [8].

The aim of this study was to survey the extent to which hospitals in Norway treat hip fracture patients according to selected criteria in the Norwegian guidelines for interdisciplinary treatment of hip fractures. In addition, we wanted, by using data from the NHFR, to map associations between this organization of treatment and 30-day mortality as well as self-reported quality of life after hip fracture surgery.

## Methods

### Study design

This study is an observational study based on data from the Norwegian Hip Fracture Register (NHFR). The NHFR has collected data from all hip fracture operations performed in Norway since 2005 [8]. Based on the Norwegian guidelines for interdisciplinary treatment of hip fractures [1] and the UK Best Practice Tariff [7], we constructed a questionnaire of nine selected criteria for what is defined as good clinical practice in a standardized clinical pathway (Table 1). We aimed for criteria which could be possible to fulfill at all hospitals treating hip fractures. Despite the fact that an orthogeriatric approach is recommended for hip fracture patients, this is yet to be established in many Norwegian hospitals. The requirement for interdisciplinary treatment was therefore defined as the presence of either a geriatrician or a pharmacist in addition to an orthopedic surgeon and a physiotherapist. In 2020, the questionnaire was sent to the NHFR’s contact persons at all 43 hospitals that operate hip fractures in Norway to survey which of these nine criteria the hospitals fulfilled. 29 hospitals (67%) answered the questionnaire. The hospitals were awarded 1 point for each criterion they met. As none of the criteria were national quality indicators in Norway, it was considered too strict to require that all criteria should be met. At the same time, it was desirable that as many criteria as possible should be fulfilled. A standardized course of treatment of good enough quality was therefore defined as a minimum of eight out of nine criteria being met (hereafter called standardized clinical pathway). The criteria were not weighted.

**Table 1:** Number of hospitals fulfilling each of the nine criteria for a standardized clinical pathway (*N* = 29)

Criteria for a standardized clinical pathway	*n*	%
Both physiotherapist, orthopedic surgeon, geriatrician, and/or pharmacist involved in treatment	22	76
Preoperative peripheral nerve block	29	100
Mobilization at day 0 or day 1 after surgery	29	100
Removal of urine catheter at day 0 or day 1 after surgery	27	93
Drug review performed by geriatrician, pharmacist or other physician	27	93
Screening for delirium	21	72
Assessment of risk of malnutrition, falls, and pressure ulcers	26	90
Initiation of anti-osteoporosis drugs	28	97
Follow-up from the hospital after discharge	16	55

### Outcomes

Data from the NHFR was used to compare outcomes for hospitals with and without a standardized clinical pathway. The NHFR contains information on age, sex, and comorbidity (American Society of Anesthesiologists (ASA) class). Data on death was provided by the National Population Register in Norway. The primary outcome was 30-day mortality. The NHFR also collects information on quality of life from hip fracture patients 4, 12, and 36 months postoperatively using the Norwegian validated version of the EQ-5D-3L [9]. The EQ-5D-3L is a standardized generic instrument for measuring health outcomes that contains five dimensions, each with three response options (the patient's health status). EQ-5D index scores were generated from a European reference population [10]. An index score of 1 represents the best possible quality of life, while an index score of 0 represents a quality of life equivalent to death.

### Statistical analysis

Categorical variables were described with numbers and percentages, and proportions were compared between groups using Pearson Chi-squared test. Student’s *t* test was used for continuous variables. 30-day mortality for hospitals with and without a standardized clinical pathway was compared using Cox regression model with adjustment for age group, sex, and ASA class. 30-day mortality at the hospitals that did not answer the questionnaire was also calculated. Patients operated on in the 5-year period from 2016 to 2020 were included in the main analyses. Since the treatment course at the hospitals may have changed during this time period, analyzes were also performed including only patients treated in the years 2019–2020. Hazard ratio (HR) with 95% confidence interval (CI) was calculated. *p* values less than 0.05 were considered statistically significant.

### Ethics

The Norwegian Hip Fracture Register has its authorization from the Norwegian Data Protection Authority to collect and store data on hip fracture patients (authorization issued on 3 January 2005: reference number 2004/1658-2 SVE/-). Approval by the Regional Ethical Committee was accordingly not required.

## Results

At the 29 hospitals that answered the questionnaire, 32,689 hip fractures were operated in the period 2016–2020. In the same period, 40,168 hip fracture operations were registered in the NHFR.

### Adherence to criteria

Table 1 shows the number and proportions of the 29 hospitals that met the nine different criteria for a standardized treatment course. A total of 20 hospitals (69%) met the requirement for a standardized clinical pathway, i.e., the hospital met at least eight of the nine selected criteria. Of these, 11 hospitals fulfilled all nine criteria and nine hospitals eight criteria. Of the remaining nine hospitals, two fulfilled seven criteria, six fulfilled six criteria and one fulfilled five criteria.

There was a minor difference in age group distribution, but no difference in average age, gender, or ASA class between patients operated at hospitals with or without a standardized clinical pathway (Table 2).

**Table 2:** Baseline characteristics for hip fracture patients treated at hospitals with or without a standardized clinical pathway in the time period 2016–2020

	Hospitals with a standardized clinical pathway^a^	Hospitals without a standardized clinical pathway^a^	Hospitals not responding to questionnaires	*p* value
Total *n*	22,732	9957	7479	
Mean age (SD)	80.0 (11.6)	80.1 (11.3)	79.7 (11.1)	0.035^b^
Age group (%)				0.002^c^
< 75	6165 (27.1)	2643 (26.5)	2131 (28.5)	
75–79	3081 (13.6)	1408 (14.1)	1064 (14.2)	
80–84	3976 (17.5)	1850 (18.6)	1350 (18.1)	
85–90	4908 (21.6)	2066 (20.7)	1534 (20.5)	
>= 90	4602 (20.2)	1990 (20.0)	1400 (18.7)	
Women (%)	15,167 (66.7)	6560 (65.9)	4985 (66.7)	0.320^c^
ASA class (%)				0.341^c^
1–2	7876 (34.6)	3433 (34.5)	2522 (33.7)	
3–5	14,856 (65.4)	6524 (65.5)	4957 (66.3)	

^a^Standardized clinical pathway: ≥ 8 out of 9 criteria met

^b^Student’s *t* test

^c^Pearson Chi-squared test

### Mortality

**Fig. 1:**
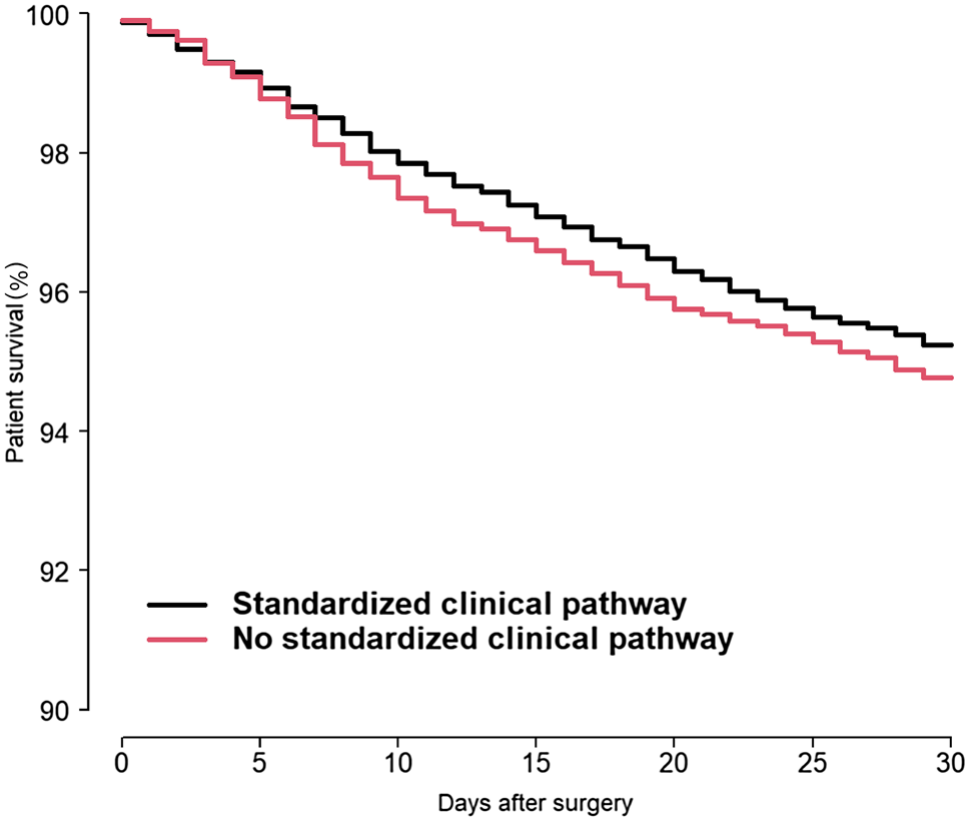
30-day mortality for patients treated in the period 2016–2020. Cox survival curve with adjustments for differences in age group, sex, and ASA class

30-day mortality was investigated for patients operated in two different time periods. For the period 2016–2020, the 30-day mortality rate was 7.6% at all hospitals, 7.3% for patients in hospitals with a standardized clinical pathway, and 8.3% for patients at hospitals without a standardized clinical pathway. Cox analysis with adjustment for age group, sex, and ASA class showed a statistically significantly higher 30-day mortality in hospitals without a standardized clinical pathway compared to hospitals with a standardized clinical pathway [HR 1.13 (1.04–1.23), *p* = 0.005] (Fig. 1). There was no difference in 30-day mortality between hospitals with a standardized clinical pathway and hospitals that did not respond to the questionnaire [HR 1.02 (0.93–1.13), *p* = 0.640].

In the period 2019–2020, 30-day mortality was 7.3% for all hospitals, 7.0% for patients in hospitals with a standardized clinical pathway and 7.8% for patients in hospitals without a standardized clinical pathway. Cox analysis with adjustment for age, sex, and ASA class showed no difference in 30-day mortality between hospitals with and without a standardized course [HR 1.10 (0.96–1.27), *p* = 0.16].

### Patient-reported outcomes

**Fig. 2:**
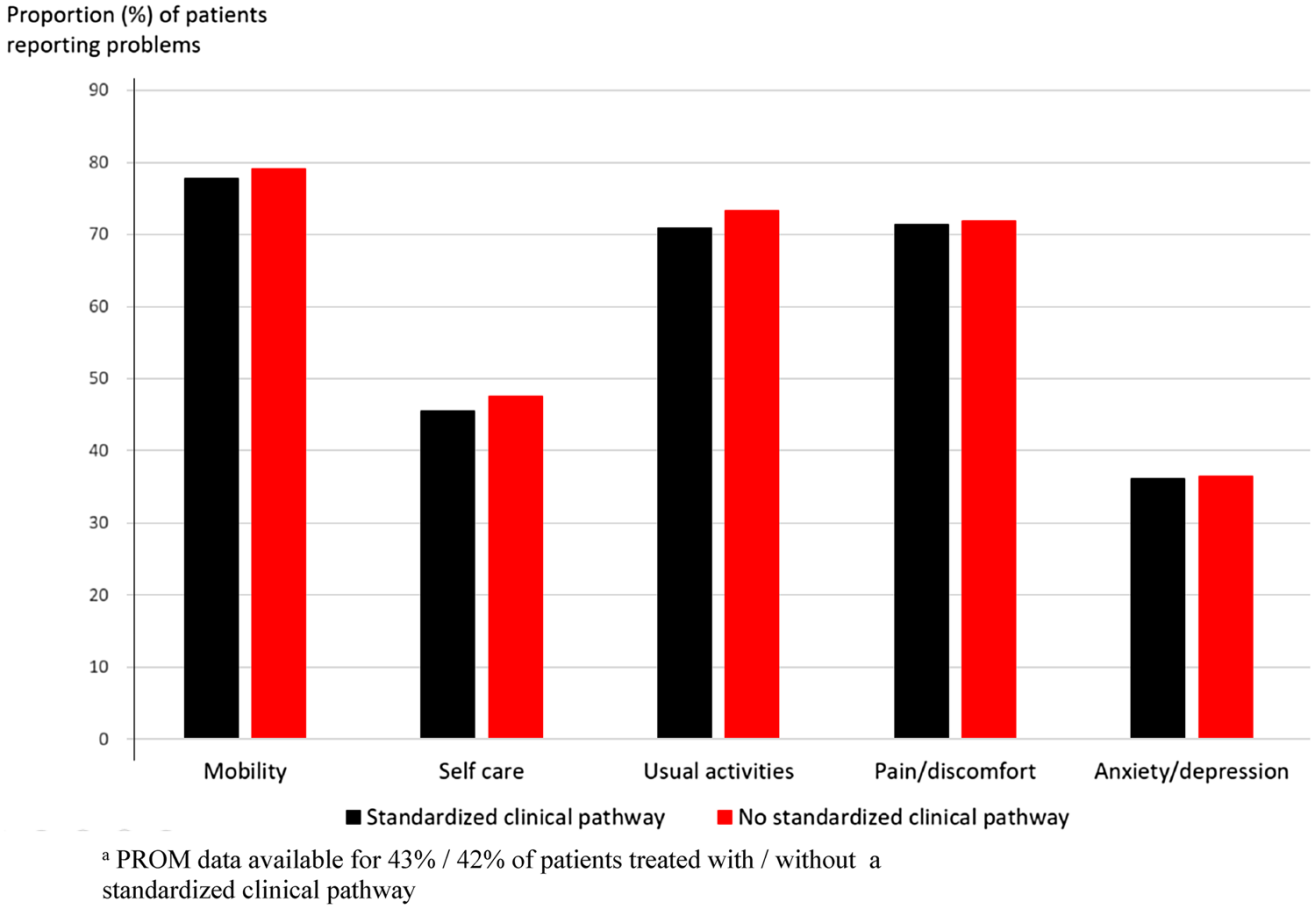
Proportion of hip fracture patients treated in 2016–2020 reporting to have some or severe problems in each of the five dimensions of EQ-5D-3L 4 months postoperatively^a^

4 months postoperatively, patients treated in hospitals with a standardized clinical pathway (*n* = 9872) and patients treated in hospitals without a standardized clinical pathway (*n* = 4177) reported an EQ-5D index score of 0.58 and 0.57 respectively (*p* = 0.038). Analyses of each of the five dimensions in the EQ-5D-3L consistently showed a slightly higher level of functioning in patients treated at hospitals with a standardized treatment course (Table 3 and Fig. 2).

**Table 3:** Descriptive profile of the five dimensions of EQ-5D-3L 4 months after hip fracture. Patients treated in 2016–2020

	Total *n*^b^ (%)	Standardized clinical pathway^a^		
		Yes^b^ (%)	No^b^ (%)	*p* value^c^
Mobility				0.187
No problems in walking about	3172 (22)	2271 (22)	901 (21)	
Some problems in walking about	10,763 (74)	7541 (74)	3222 (75)	
Confined to bed	612 (4.2)	422 (4.1)	190 (4.4)	
Self-care				0.014
No problems with self-care	7898 (54)	5623 (55)	2275 (52)	
Some problems with washing or dressing	5084 (35)	3552 (35)	1532 (35)	
Unable to wash or dress	1658 (11)	1124 (11)	534 (12)	
Usual activities				0.003
No problems with performing usual activities	4130 (28)	2978 (29)	1152 (27)	
Some problems with performing usual activities	7373 (51)	5175 (51)	2198 (51)	
Unable to perform usual activities	3041 (21)	2082 (20)	959 (22)	
Pain/discomfort				0.790
No pain or discomfort	4150 (29)	2936 (29)	1214 (28)	
Moderate pain or discomfort	9291 (64)	6521 (64)	2770 (64)	
Extreme pain or discomfort	1117 (7.7)	789 (7.7)	328 (7.6)	
Anxiety/depression				0.702
Not anxious or depressed	9268 (64)	6528 (64)	2740 (64)	
Moderately anxious or depressed	4627 (32)	3235 (32)	1392 (32)	
Extremely anxious or depressed	634 (4.4)	452 (4.4)	182 (4.2)	

^a^Standardized clinical pathway: ≥ 8 out of 9 criteria met

^b^The total numbers in each dimension may vary, as some patients did not answer all dimensions of the EQ-5D-3L questionnaire

^c^Pearson Chi-squared test

## Discussion

Only 22 out of 29 orthopedic hospitals fulfilled our requirement for a standardized clinical pathway based on our selected criteria from the Norwegian guidelines for multidisciplinary treatment of hip fractures. In the period 2016–2020, treatment at a hospital without a standardized clinical pathway was associated with a higher 30-day mortality compared to treatment at a hospital with a standardized clinical pathway. Patients treated at a hospital with a standardized clinical pathway consistently reported a slightly better level of function 4 months postoperatively, but no clinically significant difference in average patient-reported overall quality of life could be found.

Patients with hip fractures represent an old and frail population. Mortality, especially in the early postoperative period, is high. Identifying measures that can reduce mortality is therefore important. Orthogeriatric treatment has in several studies been shown to reduce both in-hospital and long-term mortality after hip fracture [11–13]. An orthogeriatric approach also results in better mobility [14, 15], more independent patients, higher likelihood of discharge directly home [16], and less use of health services in the period after a hip fracture [14]. The presence of geriatricians as part of a multidisciplinary team is central in the UK Best Practice Tariff criteria [7]. Access to geriatricians is therefore an important factor in optimizing the treatment of hip fracture patients and, accordingly, the Norwegian guidelines for interdisciplinary treatment of hip fractures recommend an orthogeriatric approach. Orthopedic surgeons, nurses, and physiotherapists are always included in the treatment of hip fracture patients in Norway, but geriatricians are lacking and much work remains to establish a multidisciplinary orthogeriatric service at many Norwegian hospitals. In this study, we could therefore not require the presence of geriatricians to fulfill the requirements for a standardized clinical pathway, but instead required the presence of either a geriatrician or a pharmacist, and thus interdisciplinary collaboration beyond the established minimum staff.

Although only 69% of the hospitals that answered the questionnaire met our requirement for a standardized clinical pathway, certain criteria were met by most of the hospitals. All the hospitals had introduced routines for peripheral nerve block as preoperative pain relief. Also, all hospitals mobilized the patient on the day of surgery or the first postoperative day, shown to increase the odds of being discharged directly home [16, 17]. Further, 97% reported having introduced routines for initiating anti-osteoporosis treatment. Anti-osteoporosis treatment has been shown to both reduce future osteoporotic fractures and mortality after hip fracture [18]. Only 22 out of 29 hospitals reported that hip fracture patients were followed up by a surgeon or a physiotherapist. This indicates a partially inadequate follow-up of hip fracture patients after transfer to the primary healthcare service and that interaction with the primary healthcare service after a hospital stay probably can be improved. The newly published world guidelines for falls prevention and management recommends that after sustaining a hip fracture, an individualized and progressive exercise program should be initiated during hospital stay and followed up in the community [19]. There was also a clear potential for improvement for routine delirium screening during the hospital stay. Delirium is a burden for both patients [20], relatives, and healthcare personnel, and is associated with increased morbidity and mortality, and increased length of stay [21]. After a hip fracture, independence in activities of daily living (ADL) is crucial for patients' quality of life. It has previously been shown that orthogeriatric intervention affects ADL after hip fracture surgery [22]. In our study, without requirements for geriatrics in a standardized clinical pathway, we found no clinically significant difference in self-reported overall quality of life. The difference between the groups in our study was only 0.01 points. The minimum clinically significant difference of EQ-5D index score has earlier been reported to be 0.06–0.08 [23, 24]. However, there was a slightly higher proportion of patients at hospitals with a standardized clinical pathway who could perform personal care and daily activities without problems. Accordingly, a standardized clinical pathway appears to influence the patients’ level of function postoperatively somewhat, and to have an impact on the degree of independence in daily life activities after a hip fracture. Hip fractures are highly prevalent among frail older people, and many lose their independence after the fracture. Thus, improvements for a limited share of the patients may potentially exert a major impact on the population level.

National guidelines are important for directing the focus on and driving the treatment offer forward [25]. There are several reasons why the treatment course at many hospitals is not good enough. Financial priorities are perhaps the most important. In the UK, hospitals’ compliance with the national guidelines for treatment of hip fractures has been measured in the National Hip Fracture Database since 2007 [26]. After the introduction of financial consequences for hospitals that do not meet the requirements (Best Practice Tariff), the proportion of patients treated with early surgery (same or next day) went from 54.5% in 2007 to 71.3% in 2011. In the same period, the 30-day mortality fell from 10.9% to 8.5% [27]. Existing national quality indicators for the treatment of hip fractures in Norway have traditionally focused on early surgery. We believe that updated quality indicators should also include other important elements in a standardized clinical pathway, such as delirium screening [20, 21, 28], presence of geriatricians [29, 30], early mobilization [16, 17], and follow-up of patients after discharge.

All hospitals treating hip fractures in Norway received the questionnaire. Of these, 29 hospitals responded, including all the largest hospitals in the country and hospitals from all health regions. This enabled us to assess the effect of a standardized clinical pathway for large parts of the country. Since 14 hospitals (18.4% of the operations) did not respond selection bias cannot be ruled out. It is hard to know how participation from these hospitals would have affected the results. We found, however, no difference in 30-day mortality between patients treated at non-included hospitals and patients treated at hospitals with a standardized clinical pathway.

In the main analysis 30-day mortality was calculated including patients treated in the time period 2016–2020. However, the responses to the survey reflected the adherence to the criteria at each hospital at the time the questionnaire was fulfilled. The course of treatment may have changed to a greater or lesser extent from the start of the time period to the time of fulfilling questionnaire in 2020. The hospitals, which at the time of the survey met the criteria for a standardized clinical pathway, have either had a good treatment course throughout the examined time period or have had a focus on improving the treatment course during the period. Although it is not clear whether the hospitals have met the requirements for a standardized clinical pathway throughout the period, a lower mortality rate was found in those hospitals which at the end of the period met these requirements. The analyses were also repeated with data from the last part of the period (2019–2020), where we can assume with greater certainty that the clinical pathways at the hospitals was unchanged. We found a similar difference between the two groups in this time period, which strengthens our results. A weakness of the study is that we do not have information on to which extent the hospitals were actually able to follow the routines and providing the clinical pathway for all their patients. In addition, other institution-related factors, such as waiting time for surgery, patient volume at the individual institution and the surgeon's level of experience, could have affected mortality after a hip fracture [31]. Finally, the response rate of the 4-month PROM questionnaire was 56%, and patient-reported outcomes were only available for around 43% of the total number of patients. One limitation of the study is that we did not have functional measures of hip function or ADL.

This study demonstrated differences in the treatment offered to hip fracture patients in Norway. A standardized clinical pathway for hip fracture patients was associated with reduced 30-day mortality, but no clinically important difference in quality of life. The results indicate that by following the national interdisciplinary guidelines, hospitals will be able to improve the treatment of hip fracture patients.

It is worrying that one out of three hospitals in this study do not have a standardized clinical pathway for hip fracture patients. There is significant potential for improvement for delirium screening and follow-up of hip fracture patients after transfer to primary care.

## Data Availability

The data of the NHFR is stored in a secure server area and is only available for a limited number of persons working in the register.

## Code availability

IBM SPSS Statistics (version 26.0; IBM Corp. Armonk, New York) for Windows and the R statistical package (http://CRAN.R-project.org) were used for the statistical analyses.

## References

[1] Ranhoff AH, Saltvedt I, Frihagen F, Raeder J, Maini S, Sletvold O (2019). Interdisciplinary care of hip fracture. Orthogeriatric models, alternative models, interdisciplinary teamwork. Best Pract Res Clin Rheumatol.

[2] Bentler SE, Liu L, Obrizan M, Cook EA, Wright KB, Geweke JF (2009). The aftermath of hip fracture: discharge placement, functional status change, and mortality. Am J Epidemiol.

[3] Bertram M, Norman R, Kemp L, Vos T (2011). Review of the long-term disability associated with hip fractures. Inj Prev.

[4] Dakhil S, Saltvedt I, Benth JŠ, Thingstad P, Watne LO, Bruun Wyller T, Helbostad JL, Frihagen F, Johnsen LG, Taraldsen K (2023). Longitudinal trajectories of functional recovery after hip fracture. PLoS One.

[5] Gjertsen JE, Dybvik E, Furnes O, Fevang JM, Havelin LI, Matre K, Engesæter LB (2017). Improved results after hip fracture surgery in Norway. Acta Orthop.

[6] Gjertsen J, Dybvik E, Kristensen TB. Nasjonalt Hoftebruddregister. Årsrapport for 2021 med plan for forbedringstiltak. Bergen 06.07.22. https://www.kvalitetsregistre.no/sites/default/files/2022-08/%C3%85rsrapport%202021%20Nasjonalt%20Hoftebruddregister.pdf

[7] Whitaker SR, Nisar S, Scally AJ, Radcliffe GS (2019). Does achieving the “Best Practice Tariff” criteria for fractures neck of femur patients improve one year outcomes?. Injury.

[8] Gjertsen JE, Engesaeter LB, Furnes O, Havelin LI, Steindal K, Vinje T, Fevang JM (2008). The Norwegian Hip Fracture Register: experiences after the first 2 years and 15,576 reported operations. Acta Orthop.

[9] Stavem K, Augestad LA, Kristiansen IS, Rand K (2018). General population norms for the EQ-5D-3 L in Norway: comparison of postal and web surveys. Health Qual Life Outcomes.

[10] Greiner W, Weijnen T, Nieuwenhuizen M, Oppe S, Badia X, Busschbach J (2003). A single European currency for EQ-5D health states. Results from a six-country study. Eur J Health Econ.

[11] Van Heghe A, Mordant G, Dupont J, Dejaeger M, Laurent MR, Gielen E (2022). Effects of orthogeriatric care models on outcomes of hip fracture patients: a systematic review and meta-analysis. Calcif Tissue Int.

[12] Grigoryan KV, Javedan H, Rudolph JL (2014). Orthogeriatric care models and outcomes in hip fracture patients: a systematic review and meta-analysis. J Orthop Trauma.

[13] Zeltzer J, Mitchell RJ, Toson B, Harris IA, Ahmad L, Close J (2014). Orthogeriatric services associated with lower 30-day mortality for older patients who undergo surgery for hip fracture. Med J Aust.

[14] Prestmo A, Hagen G, Sletvold O, Helbostad JL, Thingstad P, Taraldsen K (2015). Comprehensive geriatric care for patients with hip fractures: a prospective, randomised, controlled trial. Lancet.

[15] Watne LO, Torbergsen AC, Conroy S, Engedal K, Frihagen F, Hjorthaug GA (2014). The effect of a pre- and postoperative orthogeriatric service on cognitive function in patients with hip fracture: randomized controlled trial (Oslo Orthogeriatric Trial). BMC Med.

[16] Ferris H, Brent L, Sorenesen J, Ahern E, Coughlan T (2022). Discharge destination after hip fracture: findings from the Irish hip fracture database. Eur Geriatr Med.

[17] Sheehan KJ, Goubar A, Almilaji O, Martin FC, Potter C, Jones GD, Sackley C, Ayis S (2021). Discharge after hip fracture surgery by mobilisation timing: secondary analysis of the UK National Hip Fracture Database. Age Ageing.

[18] Lyles KW, Colón-Emeric CS, Magaziner JS, Adachi JD, Pieper CF, Mautalen C (2007). Zolendronic acid and clinical fractures and mortality after hip fracture. N Engl J Med.

[19] Montero-Odasso M, van der Velde N, Martin FC, Petrovic M, Tan MP, Ryg J, Aguilar-Navarro S, Alexander NB, Becker C, Blain H, Bourke R, Cameron ID, Camicioli R, Clemson L, Close J, Delbaere K, Duan L, Duque G, Dyer SM, Freiberger E, Ganz DA, Gómez F, Hausdorff JM, Hogan DB, Hunter SMW, Jauregui JR, Kamkar N, Kenny RA, Lamb SE, Latham NK, Lipsitz LA, Liu-Ambrose T, Logan P, Lord SR, Mallet L, Marsh D, Milisen K, Moctezuma-Gallegos R, Morris ME, Nieuwboer A, Perracini MR, Pieruccini-Faria F, Pighills A, Said C, Sejdic E, Sherrington C, Skelton DA, Dsouza S, Speechley M, Stark S, Todd C, Troen BR, van der Cammen T, Verghese J, Vlaeyen E, Watt JA, Masud T, Task Force on Global Guidelines for Falls in Older Adults (2022). World guidelines for fall prevention and management for older adults: a global initiative. Age Ageing.

[20] Instenes I, Gjengedal E, Eide LSP, Kuiper KKJ, Ranhoff AH, Norekvål TM (2018). "Eight days of nightmares …" – octogenarian patients’ experiences of postoperative delirium after transcatheter or surgical aortic valve replacement. Heart Lung Circ.

[21] Witlox J, Eurelings LS, de Jonghe JF, Kalisvaart KJ, Eikelenboom P, van Gool WA (2010). Delirium in elderly patients and the risk of postdischarge mortality, institutionalization, and dementia: a meta-analysis. JAMA.

[22] Dakhil S, Thingstad P, Frihagen F, Johnsen LG, Lydersen S, Skovlund E (2021). Orthogeriatrics prevents functional decline in hip fracture patients: report from two randomized controlled trials. BMC Geriatr.

[23] Pickard AS, Neary MP, Cella D (2007). Estimation of minimally important differences in EQ-5D utility and VAS scores in cancer. Health Qual Life Outcomes.

[24] Walters SJ, Brazier JE (2005). Comparison of the minimally important difference for two health state utility measures: EQ-5D and SF-6D. Qual Life Res.

[25] Deschodt M, Boland B, Lund CM, Saks K, Velonaki VS, Samuelsson O (2018). Implementation of geriatric care models in Europe (imAGE.eu): a cross-sectional survey in eight countries. Eur Geriatr Med..

[26] (2021) Royal College of Physicians Facing new challenges - the NHFD report on 2020 (January–December 2020). RCP, London

[27] Neuburger J, Currie C, Wakeman R, Tsang C, Plant F, De Stavola B (2015). The impact of a national clinician-led audit initiative on care and mortality after hip fracture in England: an external evaluation using time trends in non-audit data. Med Care.

[28] Pollmann CT, Mellingsæter MR, Neerland BE, Straume-Næsheim T, Årøen A, Watne LO (2021). Orthogeriatric co-management reduces incidence of delirium in hip fracture patients. Osteoporos Int.

[29] Johnsen LG, Watne LO, Frihagen F, Helbostad JL, Prestmo A, Saltvedt I (2015). Why orthogeriatrics?. Tidsskr Nor Laegeforen.

[30] Pettersen PM, Frihagen F, Saltvedt I, Figved W (2018). På tide å innføre ortogeriatri i Norge?. Tidsskr Nor Laegeforen.

[31] Kjærvik C, Stensland E, Byhring HS, Gjertsen JE, Dybvik E, Søreide O (2020). Hip fracture treatment in Norway: deviation from evidence-based treatment guidelines: data from the Norwegian Hip Fracture Register, 2014 to 2018. Bone Jt Open.

